# Higher-Dose DHA Supplementation Modulates Immune Responses in Pregnancy and Is Associated with Decreased Preterm Birth

**DOI:** 10.3390/nu13124248

**Published:** 2021-11-26

**Authors:** Christina J. Valentine, Aiman Q. Khan, Alexandra R. Brown, Scott A. Sands, Emily A. Defranco, Byron J. Gajewski, Susan E. Carlson, Kristina M. Reber, Lynette K. Rogers

**Affiliations:** 1 Department of Pediatrics, University of Arizona, Tucson, AZ 85721, USA; 2 Center for Perinatal Research, The Abigail Wexner Research Institute at Nationwide Children’s Hospital, Columbus, OH 43215, USA; Aiman.Khan@Nationwidechildrens.org (A.Q.K.); Lynette.Rogers@Nationwidechildrens.org (L.K.R.); 3 Department of Biostatistics & Data Science, University of Kansas Medical Center, Kansas City, KS 66160, USA; abrown8@kumc.edu (A.R.B.); bgajewski@kumc.edu (B.J.G.); 4 Department of Dietetics and Nutrition, University of Kansas Medical Center, Kansas City, KS 66160, USA; ssands@kumc.edu (S.A.S.); scarlson@kumc.edu (S.E.C.); 5 Department of Obstetrics and Gynecology, University of Cincinnati College of Medicine, Cincinnati, OH 45267, USA; defranee@ucmail.uc.edu; 6 Department of Pediatrics, Baylor College of Medicine, Houston, TX 77030, USA; kmreber@texaschildrens.org; 7 Department of Pediatrics, Ohio State University, Columbus, OH 43210, USA

**Keywords:** preterm birth, pregnancy, DHA, sRAGE, IL-6, Bayesian adaptive design

## Abstract

Pregnancy and parturition involve extensive changes in the maternal immune system. In our randomized, multi-site, double-blind superiority trial using a Bayesian adaptive design, we demonstrated that 1000 mg/day of docosahexaenoic acid (DHA) was superior to 200 mg/day in preventing both early preterm birth (less than 34 weeks’ gestation) and preterm birth (less than 37 weeks’ gestation). The goal of this secondary study is to compare the effects of 1000 mg/day versus 200 mg/day on maternal inflammation, a possible mechanism by which DHA may prevent preterm birth. Maternal blood samples were collected at enrollment (12–20 weeks’ gestation) and at delivery. Red blood cell DHA levels were measured by gas chromatography, and plasma concentrations of sRAGE, IL-6, IL-1β, TNFα, and INFγ were measured by ELISA. Data were analyzed for associations with the DHA dose, gestational age at birth, and preterm birth (<37 weeks). Higher baseline and lower delivery levels of maternal sRAGE were associated with a greater probability of longer gestation and delivery at term gestation. Higher-dose DHA supplementation increased the probability of a smaller decrease in delivery sRAGE levels. Higher IL-6 concentrations at delivery were associated with the probability of delivering after 37 weeks, and higher-dose DHA supplementation increased the probability of greater increases in IL-6 concentrations between enrollment and delivery. These data provide a proposed mechanistic explanation of how a higher dose of DHA during pregnancy provides immunomodulatory regulation in the initiation of parturition by influencing sRAGE and IL-6 levels, which may explain its ability to reduce the risk of preterm birth.

## 1. Introduction

Parturition is associated with leukocyte infiltration and the release of cytokines to initiate labor [1,2]. The physiological events that surround preterm parturition remain elusive but previous data support the finding that maternal inflammation is a primary contributor to preterm birth. Given the anti-inflammatory properties of docosahexaenoic acid (DHA), it has been investigated as a plausible therapeutic to prolong gestation by preventing preterm parturition [3,4]. In our previous trial comparing maternal supplementation of 200 mg versus 1000 mg of DHA per day, 200 mg was found to have little to no effect on maternal, infant, or breast milk inflammatory markers, whereas the women ingesting 1000 mg per day had significant decreases in plasma and breast milk inflammatory cytokine expression [5].

DHA is a metabolically active fatty acid that has been extensively studied in the context of nutrition, neurodevelopment, and immunology [3,6,7,8,9,10]. The incorporation of DHA into membrane phospholipids affects lipid raft formation, which further affects receptor-mediated signaling and changes in membrane fluidity [3,7,10]. Investigations have demonstrated that, unlike other omega-3 fatty acids, DHA regulates interactions between cell surface ligands and receptors, resulting in attenuated inflammation [11,12,13,14,15].

Preterm birth is associated with both low maternal blood levels of DHA and low DHA dietary intake [16,17]. Maternal DHA supplementation has demonstrated efficacy in preventing preterm birth, but the optimal dose required to achieve a protective effect has not been clearly defined and the mechanism of action has not been identified [4]. Experts recommend ingesting between 200 mg and 1000 mg of DHA each day during pregnancy [18], and prenatal vitamin supplements typically provide DHA, but most contain 200 mg or less. To address the need for evidence-based data on DHA dosing, we hypothesized that 1000 mg of DHA per day would be superior to 200 mg in preventing preterm birth [19]. Our randomized, multi-site, double-blind superiority trial using a Bayesian adaptive design demonstrated that the higher dose of DHA was superior to the lower dose in preventing both early preterm birth (less than 34 weeks’ gestation) and preterm birth (less than 37 weeks’ gestation) with participants with low baseline DHA levels at enrollment benefiting most from the higher-dose supplement [19].

Given the important role of inflammation in the preterm parturition cascade, this study tested a secondary hypothesis in this Phase 3 clinical trial: that supplementation with 1000 mg per day of DHA would be superior to 200 mg per day in regulating inflammatory responses and would provide a mechanism by which DHA prevents preterm birth. Several markers have been identified as indicative of inflammation in pregnancy [20,21,22] and some of the most significant are the focus of this investigation: the Receptor for Advanced Glycation End Products (specifically, soluble and extracellular forms, sRAGE), Interleukin (IL)-6, IL-1β, Tumor Necrosis Factor alpha (TNFα) and Interferon gamma (IFNγ). Data were analyzed for associations with the DHA dose, gestational age at birth, and preterm birth (<37 weeks).

## 2. Materials and Methods

### 2.1. Study Design

The study design and enrollment details of the parent study have been previously published [19]. Women were recruited from three large academic medical centers: the University of Kansas, Ohio State University, and the University of Cincinnati. The study was approved by the University of Kansas Medical Center IRB which granted approval under a central IRB with reliance by the other institutions (STUDY00003455).

**Figure 1:**
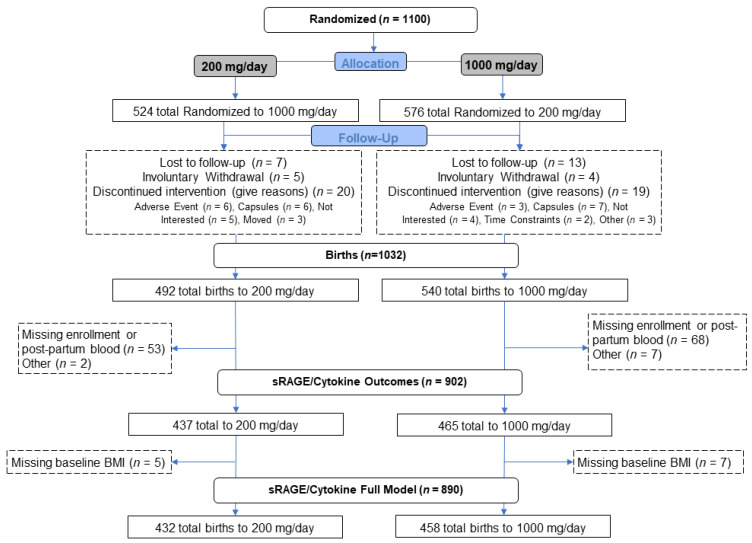
Study Design and Enrollment.

Briefly, pregnant women were randomly assigned to take 2 capsules of algal oil daily (totaling 800 mg of DHA) or soybean and corn oil (0 mg of DHA) beginning between 12 and 20 weeks of gestation. Both groups received a commercially available prenatal supplement containing 200 mg of DHA. Therefore, the experimental group received 1000 mg of DHA per day and the control group received 200 mg of DHA per day. The final enrollment for the parent study included 1100 randomized participants; 492 women in the 200 mg group and 540 in the 1000 mg group (details are provided in Figure 1). DHA levels were measured and reported in the original publication using standard fatty acid extraction and gas chromatography measurements [17,19]. The immune markers, sRAGE and cytokines, were measured in participants that provided both enrollment and delivery samples for testing. The final numbers analyzed for this study were 902 individuals; 437 who received 200 mg/day and 465 who received 1000 mg/day.

### 2.2. Blood Collection

Maternal blood samples were collected at enrollment and delivery hospitalization as previously described [19]. All samples were stored at −80 °C for the measurement of sRAGE and inflammatory cytokines.

### 2.3. ELISA

Analyses were performed on participants for whom both enrollment and delivery samples were available (*n* = 437, 200 mg per day; *n* = 465, 1000 mg/day) (Figure 1). sRAGE was measured using an ELISA-based format (MesoScale Diagnostics, Rockville, MD, USA) according to the manufacturer’s protocols and was analyzed independently. Cytokines, specifically IL-6, IL-1β, TNFα, and IFNγ, were measured on plasma samples using a similar multi-plex ELISA platform.

### 2.4. Statistical Analyses

Normal Bayesian models were used to assess the effect of the sRAGE and cytokine levels of the treatment group at enrollment or delivery on maternal serum DHA sRAGE and cytokine concentrations. Normal Bayesian models also quantified the effect of the sRAGE and cytokine levels at enrollment and delivery on the outcome of gestational age at birth in weeks while accounting for the confounding variables (treatment group, maternal race and ethnicity, BMI group, history of preeclampsia, DHA at enrollment, and smoking history (before or during pregnancy)). Similarly, binomial Bayesian models were fit for the dichotomous outcome of preterm birth (<37 weeks’ gestation, yes/no). Data are reported as posterior means and Bayesian credible intervals with posterior probabilities. The findings can be interpreted as the probability that DHA supplementation and the marker of interest are associated.

We utilized OpenBUGS version 3.2.3 rev 1012 for all Bayesian analyses (open access software developed by OpenBUGS Foundation; http:openbugs.net/w/OpenBUGS_3_2_3?Action=AttachFile&do=get&target=OpenBUGS-3.2.3.tar.gz). All analyses were fitted using 10,000 burn-in draws of Markov chain Monte Carlo, followed by 40,000 draws for inference. All prior distributions were non-informative.

## 3. Results

sRAGE and cytokine measurements were performed on all participants with available enrollment and delivery maternal blood samples. The parent study had 1032 births for which sRAGE and cytokine measurements could have been collected and analyzed. Overall, <0.01% (4/1032) were missing baseline blood samples while 11.3% (117/1032) were missing delivery blood samples. A total of 11.7% (121/1032) were unable to have their sRAGE and cytokine data analyzed due to missing either a baseline or delivery blood draw. There were also 0.9% (9/1032) with samples unusable for processing and analysis, detailed in Figure 1. For infants born prematurely, prior to 37.0 weeks’ gestation, 26.3% (31/118) were missing sRAGE and cytokine data due to missing or unusable blood samples while 10.8% (99/914) of term deliveries were missing sRAGE and cytokine data. A descriptive summary of maternal sRAGE and the cytokine concentrations as well as pregnancy characteristics are included in Table 1.

Differences in the probability of a higher dose of DHA (1000 mg per day) to prevent preterm birth compared to a lower dose (200 mg per day) were noted in the parent trial between women with high (>6% of RBC total fatty acids) baseline DHA levels versus low baseline DHA (<6% of RBC total fatty acids) levels. As previously reported, 54/492 (11%) births occurred at <37 weeks in the 200 mg/day group compared to 44/540 (8.2%) in the 1000 mg/day group, with a 0.95 posterior probability that 1000 mg was better than 200 mg [19]. Because of this finding, the baseline percent of DHA in red blood cells was included as a confounding variable. Other variables included in the analyses were smoking history (before or during pregnancy), maternal race and ethnicity (non-Hispanic Black versus other race and ethnicity), history of preeclampsia, and enrollment BMI (>30 versus ≤30 kg/m^2^). Twelve participants (5 at 200 mg/day and 7 at 1000 mg/day) were removed from the final models because data on baseline BMI were missing. The primary outcomes were gestational age at birth in weeks and preterm birth prior to 37 weeks of gestation.

As found in the parent study, treatment with 1000 mg per day demonstrated a significant posterior probability (pp = 0.99) of having a greater gestational age at birth compared to treatment with 200 mg/day (Table 2). sRAGE concentrations were associated with gestational age at birth after adjusting for group and other covariates in the full model. Mothers with a higher baseline sRAGE concentration had a significantly longer gestation (pp = 0.99). Conversely, higher delivery sRAGE levels were predictive of an earlier gestational age at birth (pp = 0.00). Race, pre-pregnancy BMI, history of preeclampsia, DHA at enrollment, and smoking history were found to be significant variables and were included in the final models. The association between sRAGE, DHA, pregnancy characteristics, and gestational age at birth are summarized in Table 2.

Additional analyses using preterm birth (<37 weeks, yes vs. no) as a binary outcome revealed similar findings (Table 2). As baseline sRAGE levels decreased, the probability of experiencing a preterm birth increased (pp = 0.92). Moreover, lower delivery sRAGE levels are associated with a decrease in the odds of preterm birth (pp = 0.002). All defined variables were included in the model.

The effects of the cytokines IL-6, IL-1β, TNFα, and IFNγ on gestational age at birth and preterm birth were analyzed individually, while adjusting for the covariates mentioned earlier (Table 3). Higher concentrations of TNFα and INFγ at delivery were associated with an earlier gestational age at birth (pp = 0.07 and pp = 0.06, respectively). No other significant associations between cytokines and gestational age at birth were found. In the binary analyses of preterm birth, higher levels of both enrollment and delivery IL-6 and lower enrollment levels of TNFα were associated with an increased probability of experiencing a birth at less than 37 weeks (pp = 0.87, 0.85, and pp = 0.03, respectively).

The relative changes (delivery concentrations minus enrollment concentrations) in sRAGE, IL-6, IL-1β, TNFα, or IFNγ were analyzed with the treatment group alone as a predictor (Table 4). A smaller decrease in sRAGE and a greater increase in IL-6 (delivery concentrations minus enrollment concentrations) were both associated with a probability that 1000 mg/day is better than 200 mg/day (pp = 0.84 and 0.99, respectively) in modulating the expression of these molecules. There was a significantly smaller decrease in sRAGE for mothers receiving the higher-dose DHA supplement (1000 mg per day) compared to those receiving the lower-dose DHA supplement (200 mg per day, pp = 0.84). The higher-dose DHA supplement group had a substantially larger increase in IL-6 from baseline to delivery compared to the lower-dose DHA supplement group (pp = 0.99).

## 4. Discussion

Modulation of the maternal immune system occurs during pregnancy to protect the fetus [23]. Inflammation is essential for the initiation of labor but has also been linked to an increased risk of developing pregnancy-related morbidities such as chorioamnionitis and preterm birth [1]. Studies in vitro associated DHA with the attenuation of transcription factor activities such as nuclear factor (NF)-kB, mitogen-activated protein (MAP) kinases, and transcriptional activation of the cytokines that are associated with inflammation, such as TNFα, IL1, and IL-17 [24]. Understanding whether these mechanisms may contribute to the prevention of preterm birth associated with higher-dose DHA supplementation is therefore important to understand.

Leukocyte infiltration, cytokine release, and the resulting inflammation contribute to the initiation of parturition, and the maternal response to this inflammation provides protective effects for both mother and infant [1,2]. RAGE is a transmembrane receptor with distinct extra- and intra-cellular domains, and full-length RAGE is membrane bound and propagates signaling pathways regulating inflammation or immune responses [25]. Alternatively, RAGE exists in multiple extracellular isoforms (soluble RAGE or sRAGE) as a result of either alternative splicing or proteolytic cleavage [26,27,28]. For the purposes of this study, the specific forms of extracellular RAGE were not distinguished, and all extracellular and soluble RAGE was measured and referred to as sRAGE. sRAGE has been described with opposing functions: first, as a decoy receptor binding ligands and preventing intracellular signaling; and second, by binding CD11b on the surface of leukocytes, activating NFkB-mediated pathways, and propagating inflammation [25,29,30]. Consequently, the function of sRAGE depends upon the extracellular milieu and the severity of the inflammatory responses. While previous studies have reported differing findings, most have described sRAGE levels decreasing from early gestation to parturition, but substantially lower sRAGE levels were observed in mothers who experience spontaneous preterm delivery. On the contrary, inflammatory conditions that are associated with medically indicated preterm birth, such as preeclampsia, have generally led to higher sRAGE levels at delivery [31,32].

Several studies identified gradual increases in maternal serum sRAGE levels through the second trimester and then significant decreases approaching birth [33,34,35,36]. Others have speculated that the microinflammation and oxidative stress that occur during parturition create ligands for RAGE and sRAGE and that available sRAGE then acts as a decoy receptor to prevent deleterious responses. This would explain the decreases in sRAGE at birth and support the concept that higher levels early in gestation are protective [32].

Mothers who give birth prematurely (<37 weeks) have lower levels of sRAGE than mothers who deliver at term [21,32,36,37]. This further decrease in sRAGE concentration is likely due to the events associated with the onset of preterm labor (or parturition) which are often associated with inflammation and similar inflammatory mediators that interact with available sRAGE. Several investigations have suggested that higher levels of sRAGE are associated with longer latency in the context of preterm birth [37], and Bastek et al. reported that lower maternal serum sRAGE levels were associated with a two-fold increase in the odds of preterm birth [38].

Our data are in line with previous reports that a higher baseline level of sRAGE offers protection against preterm birth, allowing for a buffer against pregnancy-related inflammatory responses. We observed that higher levels of sRAGE at enrollment were associated with a high probability of longer gestation and a greater probability of birth at term (≥37 weeks) (Table 2). However, in the context of morbidities such as preeclampsia and obesity, maternal inflammation occurs at a higher level throughout gestation, and sRAGE concentrations are increased to respond to this challenge. Thus, higher sRAGE levels at parturition are associated with a high probability of shorter gestational length and a greater probability of preterm birth (<37 weeks) (Table 2). Finally, smaller changes in sRAGE were associated with higher-dose DHA supplementation (1000 mg per day) compared to a standard lower dose (200 mg per day, Table 4) and a greater probability that higher-dose DHA supplementation is better than a lower dose in maintaining sRAGE levels during pregnancy. This data may be interpreted to mean that supplementing with 1000 mg per day of DHA supports a more robust production of sRAGE and the capacity to reduce inflammation in pregnancy. An important limitation to note was there was more sRAGE (and cytokine) data missing for those born prematurely compared to the term births, as noted above in Section 3. Interesting future work could incorporate Bayesian methods using the other factors included in the models to conduct multiple imputation with the missing values.

Inflammatory responses due to paracrine interactions within the pregnant uterus are essential components in the initiation of labor. Elevations in IL-6, IL-1β, and TNFα levels occur within the process of normal delivery at term, but levels are often more elevated in the context of pregnancy complications and preterm birth [39]. IL-6 is one of the most abundant and influential cytokines throughout gestation and plays a significant role in regulating the release of prostaglandins for parturition [1,39]. While IL-6 does not stimulate contractions, the onset of labor causes a 1.5-fold increase in IL-6 compared to non-pregnant women, but the increases are greater in the context of chorioamnionitis, infection, or preterm birth [1]. Furthermore, some epidemiological evidence links higher IL-6 levels to preterm birth and suggests that IL-6 levels may be a biomarker of early delivery [22]. In the current study, no association was observed between IL-6 levels at enrollment or delivery and gestational age at birth. However, the probability of giving birth after 37 weeks’ gestation was associated with higher IL-6 levels (Table 3). As indicated in Table 4, the group treated with a higher-dose DHA supplement had a greater increase in IL-6 levels compared to those who received a lower-dose DHA supplement. One interpretation of these findings is that IL-6 plays a significant role in regulating the parturition process and that higher levels support uterine quiescence until term gestational age.

IL-1β is a significant regulator of inflammation and labor-inducing genes. It is a stimulator of COX-2 and thus prostaglandin synthesis and is an upstream inducer of IL-6 production [20,22,40]. IL-1β activates and regulates early responses to infection, activates NF-kB, and is associated with the onset of spontaneous preterm birth. Our analyses did not identify any significant differences in IL-1β and gestational age at birth, preterm birth (<37 weeks), or the DHA treatment groups (Table 3 and Table 4). TNFα is a proinflammatory cytokine that promotes protection from bacterial and viral infections but has also been associated with medical complications of pregnancy such as diabetes and preeclampsia [20,22,40]. Higher TNFα levels at delivery were associated with an increased probability of a shorter gestational length and a greater probability of preterm birth (<37 weeks) (Table 3). INFγ acts as a chemoattractant and activates macrophages to facilitate host responses in the face of cellular pathogens. Higher INFγ levels at delivery were associated with the probability of an earlier gestational age at birth [20,22,39] (Table 3). Neither TNFα nor INFγ were associated with the DHA supplement amount (high versus low dose). TNFα and INFγ are not causally associated with the parturition process but are elevated with inflammatory conditions associated with pregnancy such as chorioamnionitis [40], exposure to environmental pollutants [41], preterm rupture of membranes [41], preeclampsia, gestational diabetes [22], and preterm birth [20].

## 5. Conclusions

In summary, our data indicate that higher baseline and lower delivery levels of maternal sRAGE are associated with a greater probability for longer gestation and delivery at term gestation. Furthermore, we found that higher-dose DHA supplementation increased the probability of a smaller decrease between sRAGE levels at enrollment and delivery. Higher IL-6 concentrations at delivery were also associated with the probability of delivering after 37 weeks and higher-dose DHA supplementation increased the probability of greater increases in IL-6 concentrations between enrollment and delivery. These data obtained from a prospective clinical trial provide a potential mechanistic explanation of how a higher-dose DHA supplement during pregnancy might provide immunomodulatory regulation of the initiation of parturition through its influence on sRAGE and IL-6 levels, which may explain its ability to reduce the risk of preterm birth. A prospective study to evaluate these relationships is needed.

## Figures and Tables

**Table 1:** Descriptive Summary of Variables.

	200 mg/day *n* = 437 (47.6%)	1000 mg/day *n* = 465 (52.4%)	Total *n* = 902
sRAGE [pg/mL], mean (SD)			
Enrollment	564.4 (286.4)	520.6 (279.4)	541.8 (283.5)
Delivery	467.7 (277.3)	443.1 (381.4)	455.0 (335.1)
IL 6 [pg/mL], mean (SD)			
Enrollment	0.9 (1.0)	1.3 (6.1)	1.1 (4.4)
Delivery	4.5 (7.5)	7.7 (26.0)	6.1 (19.4)
IL 1β [pg/mL], mean (SD)			
Enrollment	0.1 (0.2)	0.1 (0.2)	0.1 (0.2)
Delivery	0.2 (0.8)	0.2 (1.0)	0.2 (0.9)
TNFα [pg/mL], mean (SD)			
Enrollment	1.7 (0.7)	1.9 (2.1)	1.8 (1.6)
Delivery	2.1 (1.0)	2.1 (1.0)	2.1 (1.0)
INFγ [pg/mL], mean (SD)			
Enrollment	6.4 (30.8)	5.1 (13.5)	5.7 (23.5)
Delivery	5.1 (22.7)	4.4 (8.9)	4.7 (17.0)
DHA in RBC fatty acids [%], mean (SD)	6.5 (1.8)	6.4 (1.8)	6.5 (1.8)
Smoker (before or during pregnancy), yes *n* (%)	105 (24.0)	112 (24.1)	217 (24.1)
Race, *n* (%)			
Non-Hispanic Black	104 (23.8)	89 (19.1)	193 (21.4)
Other	333 (76.2)	376 (80.9)	709 (78.6)
History of Preeclampsia, yes *n* (%)	33 (7.6)	30 (6.5)	63 (7.0)
BMI Group, *n* (%)	*n* = 432	*n* = 458	*n* = 890
Obese	138 (31.9)	159 (34.7)	297 (33.4)
Other (BMI < 30)	294 (68.1)	299 (65.3)	593 (66.6)

**Table 2:** Posterior means (Bayesian credible interval) and Bayesian posterior probability for sRAGE concentrations and the continuous variable (gestation age at birth) or the binary variable (preterm birth). The means are slopes for gestation age at birth and log-odds ratios for preterm birth.

	Posterior Mean (95% Bayesian Credible Interval)	Bayesian Posterior Probability
**Gestational age at birth**		
Treatment 1000 vs. 200 mg per day	0.23 (0.03, 0.42)	0.99
Enrollment sRAGE [pg/mL]	0.0006 (0.0002, 0.0011)	0.996
Delivery sRAGE [pg/mL]	−0.0008 (−0.0012, −0.0004)	0.00
Significant variables		
Maternal Race (non-Hispanic Black vs. other)	−0.44 (−0.70, −0.19)	0.0003
Pre-pregnancy BMI (obese vs. other)	−0.50 (−0.73, −0.27)	0.00
History of preeclampsia (yes vs. no)	−1.17 (−1.56, −0.78)	0.00
DHA at Enrollment [%]	0.08 (0.02, 0.13)	0.996
Smoker (before or during pregnancy, yes vs. no)	−0.21 (−0.44, 0.03)	0.04
*^,^# Preterm birth (<37 weeks)		
Treatment, 1000 vs. 200 mg per day	0.59 (0.33, 0.96)	0.98
Enrollment sRAGE [pg/mL]	0.999 (0.988, 1.0)	0.92
Delivery sRAGE [pg/mL]	1.001 (1.0, 1.002)	0.002
Significant variables		
Maternal Race (non-Hispanic Black vs. other)	1.77 (0.95, 3.03)	0.04
Pre-pregnancy BMI (obese vs. other)	1.60 (0.88, 2.71)	0.07
History of preeclampsia (yes vs. no)	4.15 (1.91, 7.64)	0.0003
DHA at Enrollment [%]	0.84 (0.70, 0.99)	0.98
Smoker (before or during pregnancy, yes vs. no)	1.40 (0.75, 2.34)	0.16

At least 5000 burn-in and 40,000 Markov chain draws were performed. DHA, % total fatty acids; sRAGE pg/mL. * Modeling the probability of experiencing a preterm (<37 week) birth. # Mean (SD) gestation age at birth for all samples analyzed, 38.8 (1.6) weeks and % preterm birth (9.6%).

**Table 3:** Posterior means (Bayesian credible interval) and Bayesian posterior probability for cytokine concentrations and the continuous variable (gestation age at birth) or the binary variable (preterm birth). The means are slopes for gestation age at birth and log-odds ratios for preterm birth. For brevity, the maternal confounder parameter estimates are not shown.

	Posterior Mean (95% Bayesian Credible Interval)	Bayesian Posterior Probability
**Gestational age at birth**		
		
Enrollment IL-6 [pg/mL]	0.003 (−0.022, 0.028)	0.58
Delivery IL-6 [pg/mL]	−0.0005 (−0.0063, 0.0055)	0.44
		
Enrollment IL-1β [pg/mL]	−0.18 (−0.61, 0.25)	0.20
Delivery IL-1β [pg/mL]	−0.03 (−0.14, 0.07)	0.26
		
Enrollment TNFα [pg/mL]	0.02 (−0.04, 0.09)	0.76
Delivery TNFα [pg/mL]	−0.08 (−0.19, 0.02)	0.07
		
Enrollment INFγ [pg/mL]	−0.001 (−0.005, 0.003)	0.27
Delivery INFγ [pg/mL]	−0.005 (−0.010, 0.001)	0.06
		
* Preterm birth **(<37 weeks)**		
		
Enrollment IL-6 [pg/mL]	0.89 (0.67, 1.04)	0.87
Delivery IL-6 [pg/mL]	0.99 (0.95, 1.01)	0.85
		
Enrollment IL-1β [pg/mL]	1.53 (0.56, 3.00)	0.20
Delivery IL-1β [pg/mL]	0.91 (0.60, 1.16)	0.73
		
Enrollment TNFα [pg/mL]	0.73 (0.45, 1.04)	0.95
Delivery TNFα [pg/mL]	1.33 (1.00, 1.74)	0.03
		
Enrollment INFγ [pg/mL]	0.99 (0.95, 1.01)	0.83
Delivery INFγ [pg/mL]	0.996 (0.972, 1.010)	0.62

At least 5000 burn-in and 40,000 Markov chain draws were performed. All models included treatment, race, BMI group, history of preeclampsia, DHA at enrollment, and smoking history. The estimates were similar, and conclusions did not change from the sRAGE analysis above. * Modeling the probability of experiencing a preterm (<37 week) birth.

**Table 4:** Change in sRAGE/cytokine levels between enrollment and delivery, posterior means of the change (Bayesian credible intervals), and Bayesian posterior probability with treatment group (alone) as a predictor.

	Observed Difference (Delivery-Enrollment)		Posterior Mean (95% Bayesian Credible Interval)		Bayesian Posterior Probability (1000 mg Is Greater than 200 mg)
	200 mg	1000 mg	200 mg	1000 mg	
sRAGE [pg/mL]	−96.76 (244.43)	−77.49 (324.63)	−96.94 (−663.9, 472.4)	−77.55 (−651.5, 496.7)	0.84
IL-6 [pg/mL]	3.66 (7.33)	6.36 (23.91)	3.65 (−31.60, 39.08)	6.36 (−29.18, 42.01)	0.99
IL-1β [pg/mL]	0.15 (0.84)	0.18 (1.05)	0.15 (−1.73, 2.03)	0.18 (−1.72, 2.08)	0.70
TNFα [pg/mL]	0.39 (0.71)	0.28 (2.14)	0.39 (−2.78, 3.57)	0.28 (−2.93, 3.5)	0.16
INFγ [pg/mL]	−1.32 (38.29)	−0.66 (16.19)	−1.34 (−58.62, 56.53)	−0.67 (−58.37, 56.98)	0.64

At least 5000 burn-in and 40,000 Markov chain draws were performed.

## Data Availability

The availability of deidentified data from this study is contingent on a signed data access agreement of the participants and the PIs and approval of the planned use of the data. A specific request made to S.E.C. (scarlson@kumc.edu) or B.J.G. (bgajewski@kumc.edu) is required to generate a data output for other investigators.
